# Micro‐aerobic production of isobutanol with engineered *Pseudomonas putida*

**DOI:** 10.1002/elsc.202000116

**Published:** 2021-03-13

**Authors:** Andreas Ankenbauer, Robert Nitschel, Attila Teleki, Tobias Müller, Lorenzo Favilli, Bastian Blombach, Ralf Takors

**Affiliations:** ^1^ Institute of Biochemical Engineering University of Stuttgart Stuttgart Germany; ^2^ Microbial Biotechnology Campus Straubing for Biotechnology and Sustainability Technical University of Munich Straubing Germany

**Keywords:** 2‐ketogluconic acid, micro‐aerobic, isobutanol, NADPH, *Pseudomonas putida*

## Abstract

*Pseudomonas putida* KT2440 is emerging as a promising microbial host for biotechnological industry due to its broad range of substrate affinity and resilience to physicochemical stresses. Its natural tolerance towards aromatics and solvents qualifies this versatile microbe as promising candidate to produce next generation biofuels such as isobutanol. In this study, we scaled‐up the production of isobutanol with *P. putida* from shake flask to fed‐batch cultivation in a 30 L bioreactor. The design of a two‐stage bioprocess with separated growth and production resulted in 3.35 g_isobutanol_ L^–1^. Flux analysis revealed that the NADPH expensive formation of isobutanol exceeded the cellular catabolic supply of NADPH finally causing growth retardation. Concomitantly, the cell counteracted to the redox imbalance by increased formation of 2‐ketogluconic thereby providing electrons for the respiratory ATP generation. Thus, *P. putida* partially uncoupled ATP formation from the availability of NADH. The quantitative analysis of intracellular pyridine nucleotides NAD(P)^+^ and NAD(P)H revealed elevated catabolic and anabolic reducing power during aerobic production of isobutanol. Additionally, the installation of micro‐aerobic conditions during production doubled the integral glucose‐to‐isobutanol conversion yield to 60 mg_isobutanol_ g_glucose_^–1^ while preventing undesired carbon loss as 2‐ketogluconic acid.

**Abbreviations**
2‐KG: 2‐ketogluconic acid
KIV: 2‐ketoisovalerate

## 1 INTRODUCTION

The global market for isobutanol is anticipated to grow by 6.4% annually reaching USD 1.6 billion in 2027 (reportsanddata.com/press‐release/global‐isobutanol‐market). Being mainly produced by petrochemical synthesis, isobutanol primarily serves as chemical intermediate (46.5% in 2019) in particular for coating solvents or butyl rubber [1]. Besides, isobutanol can be used as drop‐in additive for gasoline and serves as precursor to produce aviation fuel. The C4 alcohol exhibits a higher combustion power, a lower vapor pressure, and a lower corrosive impact compared to ethanol [1, 2]. However, regarding the need to reduce the human carbon footprint by establishing a circular economy, industry seeks for bio‐based isobutanol production from renewable feedstocks. Accordingly, microbial isobutanol production from glucose has been established in several organisms [3, 4] such as *Escherichia coli* [5, 6], *Bacillus subtilis* [7], and *Corynebacterium glutamicum* [8, 9] under aerobic or anaerobic conditions. Nevertheless, the toxicity of isobutanol to these organisms is a major drawback preventing their use for industrial scale production with even further improved key performance values [4, 10].

Therefore, bacterial organisms such as *Pseudomonas putida* with a natural tolerance towards toxic compounds and alcohols like butanol and ethanol could be an alternative production host [11, 12, 13]. The Gram‐negative soil bacterium *P. putida* KT2440, certified as HV1 [14] is genetically accessible [15] and endowed with a versatile metabolism that originates from its ability to colonize harsh environments [16, 17]. Moreover, *P. putida* exhibits a broad range of substrate affinity which is essential for the consumption of complex harvest residuals such as lignocellulose and oils [18, 19]. The cyclic nature of glucose oxidation via the combined EDEMP pathway [20, 21] allows *P. putida* to adjust NADPH formation, a trait that is beneficial to counteract oxidative stress. As recently shown, *P. putida* is also well‐endowed to withstand transient substrate starvation conditions that may occur in large‐scale bioreactors thanks to the rapid access to intracellular 3‐hydroxyalkanoate storage buffers [22].

**PRACTICAL APPLICATION**

To meet the steadily growing demand for isobutanol as chemical precursor or fuel additive, biotechnological production approaches from renewable feedstocks are in the focus of research and industry. In contrast to bacterial strains that are sensitive to extracellular alcohol, *Pseudomonas putida* exhibits a natural tolerance towards solvents and other toxic chemicals. Therefore, we exploited this superiority with the previously engineered *P. putida* Iso2 strain to achieve high isobutanol titers with different fed‐batch strategies. By installing a micro‐aerobic production environment in the bioreactor, the balance between cellular NADPH formation and demand was improved resulting in enhanced isobutanol yields. Since carbon uptake and intracellular accumulation of succinic acid was observed during oxygen depletion, this study paves the way for innovative micro‐aerobic applications using the strict aerobic microbe *P. putida*.

These metabolic advantages render *P. putida* a suitable microbial platform for isobutanol production which was successfully shown in shake flask cultures [23]. The authors engineered the *P. putida* GN346 mutant [13], that is unable to degrade n‐butanol, for the overproduction of isobutanol from glucose via the NADPH dependent Ehrlich pathway. Besides isobutanol, high accumulation of the by‐product 2‐ketogluconic acid (2‐KG) (>0.4 g_2‐KG_ g_glc_^‐1^) was observed for *P. putida* Iso2 and engineered derivatives [23]. The problem of unwanted 2‐KG formation also occurred with *P. putida* engineered for *cis,cis*‐muconic acid production [24, 25]. *P. putida* KT2440 is known to oxidize glucose to gluconate (glt) and to 2‐KG in the periplasm via glucose dehydrogenase (Gcd) and gluconate dehydrogenase (Gad) [21, 26]. However, the deletion of *gcd* in the isobutanol producer *P. putida* Iso2 not only stopped 2‐KG production but isobutanol formation, too [23].

Since anaerobic conditions improved isobutanol production in *E. coli* [6] and *C. glutamicum* [9] through increased supply of NADPH, Nitschel et al. [23] tested *P. putida's* capability of converting glucose into isobutanol during micro‐aerobic conditions in sealed shake flasks. The authors observed minimal metabolic activity even though *P. putida* KT2440 is not capable of anaerobic respiration to control its energy and redox balance in the absence of oxygen [15, 27]. Different studies already aimed to enable *P. putida* for anoxic carbon conversion, e.g. by knocking‐in a fermentative pathway [28] or by using a redox mediator in a bioelectrochemical system [29, 30]. However, none of these attempts resulted in anaerobic growth which would require sophisticated strain engineering [31]. Further advances were achieved by creating an anaerotolerant *P. putida* strain that showed growth under micro‐oxic conditions [32]. Our study focuses on the scale‐up of isobutanol production with *P. putida* from shake flask to fed‐batch cultivations in a 30 L stirred tank reactor. Aerobic and micro‐aerobic conditions are installed to evaluate the supply of the critical electron donor NADPH as a key to improve substrate‐to‐isobutanol yields. Additionally, the cellular ATP management is quantified.

## 2 MATERIALS AND METHODS

### 2.1 Strain and medium

The *P. putida* Iso2 strain that was engineered for isobutanol production from glucose [23] was applied in all experiments. The strain is based on the GN346 variant [13] of *P. putida* KT2440 with additional deletions of *bkdAA* and *sthA*. Furthermore, it harbours the arabinose inducible plasmid pIP02 (pNG413 *araC* P_BAD_ *kivD yqhD alsS ilvC ilvD*) for overexpression of the Ehrlich pathway and the route from pyruvate to 2‐ketoisovalerate (KIV) as part of the branched‐chain amino acid pathway. The cultivation medium was adopted from Vallon et al. [33] and Davis et al. [34] and contained (per liter): 4.7 g (NH_4_)_2_SO_4_, 0.8 g MgSO_4_ · 7 H_2_O, 0.04 g CaCl_2_ · 2 H_2_O, 0.5 g NaCl, 4 g KH_2_PO_4_; and trace elements (per liter): 4 mg ZnSO_4_ · 7 H_2_O, 2 mg MnCl_2_ · 4 H_2_O, 30 mg Na_3_C_6_H_5_O_7_ · 2 H_2_O, 2 mg CuSO_4_ · 5 H_2_O, 0.04 mg NiCl_2_ · 6 H_2_O, 0.06 mg Na_2_MoO_4_ · 2 H_2_O, 0.6 mg H_3_BO_3_, 20 mg FeSO_4_ · 7 H_2_O (Merck, Darmstadt, Germany). 50 mg_apramycin_ L^–1^ were added to ensure plasmid selection.

### 2.2 Cultivation

The seed train for each bioreactor fermentation was started with a preculture that was inoculated from the working cell bank (33% glycerol stock, stored at −70°C). The first preculture was cultivated in a 500 mL baffled shake flask containing 50 mL of minimal medium (pH 7) with 6 g glucose, 10 g 3‐morpholino‐propanesulfonic acid (MOPS), and 0.5 g yeast extract (VWR International, Radnor, Pennsylvania) per liter. After approx. 10 h of incubation (130 rpm, 30°C), cells from the first preculture were used to inoculate the second preculture in a 5 L shaking flask containing 500 mL minimal medium with 6 g_glucose_ L^−1^ and 10 g_MOPS_ L^−1^. After incubation for 14 h, the total volume of the second preculture (about 0.8 g_X_ L^–1^) was used to start batch cultivation in a 30 L stirred tank reactor (STR) (Bioengineering, Wald, Switzerland) containing 8 L minimal medium (inoculum included) with 15 g_glucose_ L^−1^, at 30°C with total pressure of 1.5 bar. The initial aeration was started at 1 L min^−1^ and increased stepwise to 8 L min^–1^ during the batch phase. Also, the initial stirrer speed of 300 min^–1^ was adjusted to maintain the dissolved oxygen (DOT) above 15%. During fermentation, the pH was controlled at 6.9 using 25% NH_4_OH (Carl Roth, Karlsruhe, Germany). After initially supplied glucose was consumed an exponential feed consisting of 600 g glucose and 12.5 g MgSO_4_ per liter controlled the biomass growth rate at a predefined value. Additionally, five‐fold trace elements were supplemented during the fed‐batch phase. Peristaltic pumps (120U, Watson‐Marlow GmbH, Rommerskirchen, Germany) were used for liquid feeding. Furthermore, the plasmid was induced by adding 1.57 g_arabinose_ L^–1^ to the medium. Three different process strategies were performed in this study comprising a reference process (*Process R*), a process with fast growing cells (*Process F*) and a process with immediate micro‐aerobic condition after plasmid induction (*Process MA*). The micro‐aerobic environment (DOT < 1%) was installed by switching from submerse aeration to head‐space aeration. Striped‐out isobutanol was trapped from the exhaust gas in 10 L deionized water (adopted from Inokuma et al. [35]). This gas washing setup yielded an isobutanol recovery of 96.3% over 12 h in a reference experiment. The O_2_ and CO_2_ volume fractions in the off gas was determined with a combined O_2_/CO_2_ gas sensor (BlueSens Gas Sensor GmbH, Herten, Germany).

### 2.3 Determination of biomass and extracellular metabolites

4 × 1 mL of biosuspension was centrifuged with 20,000 g for 5 min at 4°C, washed twice with demineralized water, transferred into pre‐weighed glass vials (1.5 mL, VWR International, Radnor, Pennsylvania) and eventually dried at 105°C for 24 h. The weight of the remaining biomass was determined using a micro balance (XP26 Delta Range®, Mettler Toledo, Gießen, Germany). The organic acids, glucose, and gluconate in the supernatant were quantified using enzyme kits (r‐biopharm AG, Darmstadt, Germany). 2‐ketogluconic acid, 2‐ketoisovalerate, and isobutanol were measured using an isocratic HPLC equipped with the RI detector (1200Series, Agilent, Santa Clara, CA, USA) and a Rezex ROA‐Organic Acid H^+ ^(300 × 7.8 mm) column (Phenomenex, Aschaffenburg, Germany) at 50°C. 0.4 mL min^−1^ of 5 mM H_2_SO_4_ was used as mobile phase for the separation of the HPLC analytes. The extracellular concentrations of l‐valine, l‐isoleucine, and l‐leucine were determined according to the amino acid detection protocol described by Buchholz et al. [36].

### 2.4 LC‐MS based analysis of intracellular metabolites

Targeted metabolome analyses of intracellular *P. putida* extracts were based on previous HILIC‐ESI‐MS studies [37, 38]. The inactivation of samples and preparation of cellular extracts was performed according following adapted procedure: 0.5 mL of biosuspension were directly mixed to 1.5 mL pre‐cooled (‐40°C) quenching solution (60% methanol (v/v), 133 mM ammonium acetate/pH 9.2) and immediately centrifugated for 45 s at 20,000 g in a precooled rotor (‐20°C). Cell pellets were frozen in liquid nitrogen and stored at ‐70°C. The frozen pellets were resuspended in pre‐cooled (‐20°C) extraction buffer consisting of methanol (66% v/v), ammonium acetate (100 mM/pH 9.2), l‐norvaline (0.15 mM), and 3‐mercaptopropionic acid (2.5 mM). Added volumes were adjusted to achieve constant biomass concentrations (c_X_ = 10–15 g L^–1^). During resuspension, sample temperature was kept below ‐20°C by rotational vortexing and chilling in a constantly cooled cryostat (‐40°C). Subsequently, the same volume of pre‐cooled chloroform (‐20°C) was added and mixed properly. Next, the samples were incubated for 1 h at ‐20°C and 1 h at room temperature in a rotary overhead‐shaker and remaining cell debris were separated by centrifugation (10 min at 20,000 g and 4°C). The upper H_2_O/methanol phase (polar metabolites) was stored at ‐70°C until measurement.

Measurements were performed on an Agilent 6410B Triple‐Quad LC‐MS/MS system. Sample preparation and chromatographic separation by alkaline polymer‐based zwitterionic hydrophilic interaction chromatography (ZIC‐pHILIC) were performed as previously described [37]. Central carbon metabolites were analyzed with high selectivity in multiple reaction monitoring (MRM) mode with high selectivity and pre‐optimized precursor‐to‐product ion transitions with a mass resolution of 0.1 u and adapted MS/MS parameters [38]. Pyridine nucleotide factors were analyzed with higher sensitivity in selective ion monitoring (SIM) mode with a mass resolution of 0.3 u and analog MS parameters. System control, acquisition, and analysis of data were performed by usage of commercial MassHunter B.06.00 software.

### 2.5 Calculation of ATP formation and demand

The total ATP generation of the cell is based on the direct phosphorylation on substrate level q_ATP,S_ (Equation 2) via carbon uptake q_S,in_ (Equation 1) and on the oxidative phosphorylation via the respiratory chain q_ATP,O_ (Equation 3). According to recent published carbon fluxes [39], a net formation of 2.67 mol_ATP_ mol_glucose_^–1^ via direct phosphorylation is assumed from glucose based carbon uptake in *P. putida*. The P/O ratio (1.33 mol_ATP_ mol_oxygen_^–1^ [40]) links the oxygen uptake to the ATP formation via oxidative phosphorylation through the electron transport chain and the proton gradient that fuels the ATP synthase. Electrons to reduce oxygen in *P. putida* cannot only be transferred by NADH or FADH_2_ but also via pyrroloquinoline (PQQ) by oxidation of glucose to gluconate and 2‐ketogluconic acid [41]. In accordance, the electron flux via glucose oxidation to 2‐KG was shown in anaerobically cultivated *P. putida* F1 [29]. The authors illustrated that the incomplete oxidation of glucose to 2‐KG is an alternative route for *P. putida* to generate ATP (q_ATP,alt_). The enzyme complex in the electron transport chain creates a proton gradient through the electron flux which drives the ATP synthase. 2 e^–^ are released per molecule of glt and 4 e^–^ per molecule of 2‐KG formed from glucose that can be used to finally reduce oxygen (Equation 4). In contrast to the ATP generation, the total growth related ATP demand can be determined by Equation 5 [40].

(1) $$q_{S,in}=q_{S}\;-q_{glt}-q_{2KG}\;$$

(2) $$q_{ATP,S}=2.67*q_{S,in}\;$$

(3) $$q_{ATP,O}=q_{O2}\;*2*P/O$$

(4) $$q_{ATP,alt}=q_{2KG}\;*2*P/O+q_{glt}*P/O$$

(5) $$\begin{array}{ccc} q_{ATP} & = & \;\mu *85\;mmol_{ATP}*g_{X}^{-1}\\ & & +\;3.96\;mmol_{ATP}*g_{X}^{-1}*h^{-1} \end{array}$$

### 2.6 In silico calculation of NADPH demand

The total NADPH supply was estimated using the ratio of 1.7 mol_NADPH _mol_glucose_^‐1^, and a NADPH surplus of 14 to 21% was assumed for growth on glucose [21, 39, 42]. The additional NADPH demand in the isobutanol production pathway was calculated based on the extracellular uptake and formation rates of KIV, l‐valine and isobutanol. If uptake of KIV was detected, it was assumed to serve as precursor for isobutanol and l‐valine. Furthermore, the intracellular flux distribution from KIV to l‐valine and to isobutanol was estimated to mirror the ratio of secreted isobutanol per l‐valine during aerobic production. During micro‐aerobic condition, no growth occurred, and stationary conditions were assumed. Then, NADPH demands for isobutanol production and 2‐KG uptake via KguD should equal NADPH formation via the enzymes Zwf, Icd and MaeB. Recently published carbon fluxes [39] and the following stoichiometries were used for carbon and NADPH balancing:

$$G6P+NADP^{+}\to 6PG+NADPH$$

$$2KG+NADPH\to 6PG+NADP^{+}$$

$$\begin{array}{ccc} Isocitrate & + & NADP^{+}\to 2-oxoglutarate+CO_{2}\\ & + & \;NADPH \end{array}$$

$$Malate+NADP^{+}\to pyruvate+CO_{2}+NADPH$$

$$2\;pyruvate+NADPH\to KIV+CO_{2}+NADP^{+}$$

KIV+glutamate→Lvaline+2−oxoglutarate

$$2-\mathrm{oxog}\mathrm{luta}\mathrm{rate}+\;\mathrm{NADPH}\to \mathrm{glut}\mathrm{amate}+\;\mathrm{NAD}P^{+}$$

$$KIV+NADPH\to isobutanol+CO_{2}+NADP^{+}$$

## 3 RESULTS

### 3.1 Scale‐up of microbial isobutanol production with *P. putida* Iso2

**FIGURE 1:**
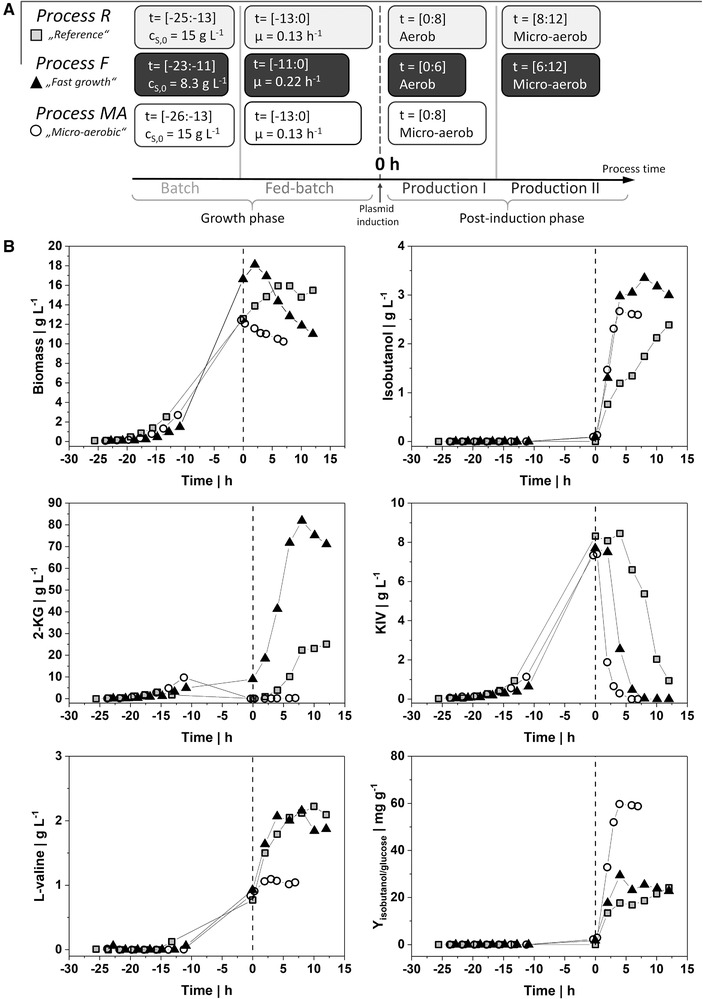
(A) Schematic overview of the three different process strategies applied in this study. *Process R* serves as reference process. After the batch and fed‐batch phase, plasmid was induced and isobutanol production was monitored. After 8 h, submerse aeration was switched to headspace aeration. In contrast, fast growing cells were induced in *Process F* and micro‐aerobic condition was installed after 6 h. Additionally, constant micro‐aerobic condition was installed in *Process MA* 20 min after plasmid induction. The time span of each phase is given in brackets. (B) Time course of biomass concentration, extracellular concentrations of isobutanol, 2‐KG, l‐valine, 2‐ketoisovalerate (KIV), and isobutanol per glucose yield Y_isobutanol/glucose_. Each plot displays the reference *Process R* (grey square), the *Process F* (black triangle), and one replicate of the *Process MA* (circle). The process time (h) is given in relation to the induction of the plasmid (dotted vertical line)

To increase biomass concentration and to monitor the growth and production performance of the metabolically engineered *P. putida* Iso2 strain [23] producing the non‐native metabolite isobutanol, the cultivation process was scaled‐up from shake flask to a 30 L stirred tank bioreactor. Growth and production were separated in two stages to analyse the effect of plasmid encoded overexpression of the isobutanol pathway on the phenotype and metabolism. Cells were cultivated under controlled conditions in the bioreactor with 8 L of initial working volume. Three different process strategies were performed. As illustrated in Figure 1A, each process consists of a batch and fed‐batch phase (*growth phase)* and a post‐induction phase (*production phase*). Figure 1B shows the time course of different product concentrations and the isobutanol yield with respect to the time of plasmid induction (*t* = 0h). The reference process is entitled as “*Process R*” and was accomplished in the following manner: During the batch process (until ‐13 h), cells grew with a maximum specific growth rate of 0.29 ( ± 0.03) h^–1^ showing a biomass specific yield of 0.16 g_X_ g_glucose_^–1^ (Table 1) and accumulated 1.7 g_2‐KG_ L^–1^_._ In the subsequent glucose limited fed‐batch phase (from ‐13 to 0 h) cells grew with μ_fb_ = 0.13 h^–1^. Cell density reached 12 g_X_ L^–1^ and 8.3 g L^–1^ of the precursor 2‐ketoisovalerate (KIV) for the isobutanol pathway accumulated during the fed‐batch phase. The feed profiles, glucose concentrations, and DOT levels are shown in the Figure S1. Next, the inducer arabinose (1.57 g L^–1^) was added to the broth to initiate the production of isobutanol (*t* = 0h). During *production phase I*, extracellular isobutanol accumulated to 1.7 g_isobutanol_ L^–1^, but the growth rate decreased to 0.02 h^–1^ within 8 h. Concomitantly, cells started to accumulate 22 g_2‐KG_ L^–1^, and 2.1 g_L‐valine_ L^–1^. Extracellular KIV concentrations remained for the first 4 h of production but declined afterwards due to cellular consumption. In *production phase II* (from 8 to 12 h), the submerse aeration was switched to head‐space aeration to install micro‐aerobic conditions. The resulting oxygen deprivation led to only minimal secretion of 2‐KG and l‐valine while the isobutanol titer increased further from 1.7 to 2.4 g L^–1^ which was accompanied by consumption of KIV. In total, 943 g glucose were consumed to produce 23.1 g isobutanol within 38 h leading to a yield of 22 mg_isobutanol_ g_glucose_^–1^ (refer to Table 1).

**TABLE 1:** Comparison of process parameters from the reference *Process R*, the *Process F* with fast growing cells and the micro‐aerobic *Process MA*

Process strategy	μ_max_ [h^–1^]	Y_X/S,max_ [g g^–1^]	μ_fed‐batch_ [h^–1^]	Y_X/S,fed‐batch_ [g g^–1^]	Y_2‐KG/S_ [g g^–1^]	Y_P/S_ [g g^–1^]	q_P,max_ [mmol g^–1^ h^–1^]
*Process R*	0.29 ± 0.03 (4)	0.16 ± 0.01 (4)	0.13 ± 0.01 (3)	0.32 ± 0.03 (3)	0.254	0.025	0.39
*Process MA*					**0.003**	**0.060**	**1.27**
*Process F*			0.22	0.37	0.623	0.022	0.65

Yields are given as product formed per glucose consumed. Standard deviation and number of replicates (in brackets) are shown after each value. Bold numbers indicate the best results. Since the batch phase was comparable in *Process R, Process F*, and *Process MA*, the mean values for μ_max_ and Y_X/S,max_ are shown. The same applies fort the comparable fed‐batch phase in *Process R* and *Process MA*.

**FIGURE 2:**
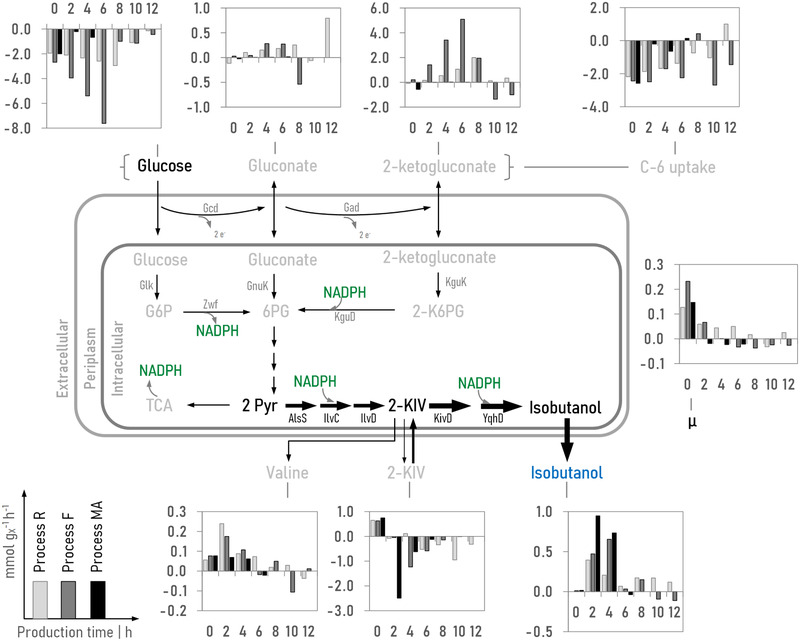
Uptake (negative) rates of carbon sources and formation (positive) rates of products (in mmol g_X_^–1^ h^–1^) as well as growth rates during production of isobutanol in the reference *Process R* (grey bar), in *Process F* (dark grey bar) and in one replicate of *Process MA* (black bar). Time point 0 h equals the reference condition before induction of the plasmid. Micro‐aerobic condition was installed in *Process R* 8 h, in *Process F* 6 h, and in *Process MA* 20 min after plasmid induction. Abbreviations (coding genes are given in parentheses): C‐6: difference of glucose uptake and gluconate and 2‐ketogluconate formation, G6P: glucose‐6‐phosphate, 2‐K6PG: 2‐keto‐6‐phosphogluconate, 6PG: 6‐phosphogluconate, Gcd: glucose dehydrogenase (*gcd*), Gad: gluconate 2‐dehdyrogenase complex (PP3382‐PP3384, PP3623, PP4232), Glk: glucokinase (*glk*), Zwf: glucose‐6‐phosphate 1‐dehydrogenase (*zwf‐1, zwf‐2, zwf‐3*), GnuK: gluconate kinase (*gnuK*), KguD: 2‐6‐phosphoketogluconate reductase (*kguD*), KguK: 2‐ketogluconate kinase (*kguK*), AlsS: acetolactacte synthase (*ilvHI/alsS*), IlvC: ketol‐acid reductoisomerase (*ilvC*), IlvD: dihydroxyacid dehydratase (*ilvD*), KivD: alpha‐ketoisovalerate decarboxylase (*kivD*), Yqhd: aldehyde reductase (*yqhD*)

Figure 2 illustrates the uptake and formation rates of the substrates and products in all three process strategies before (0 h) and after plasmid induction (2‐12 h). In the reference *Process R*, the biomass specific glucose consumption rate rose from 1.95 to 2.95 mmol_glucose_ g_X_^–1^ h^–1^ during *production phase I* even though the growth rate declined from 0.13 to 0.02 h^–1^. Simultaneously, the 2‐KG production rate increased significantly to 2.0 mmol_2‐KG_ g_X_^–1^ h^–1^ which led to a persistent decline of carbon uptake (total of C‐6 sugars). Nevertheless, the maximum isobutanol production rate of 0.39 mmol_isobutanol_ g_X_^–1^ h^–1^ was observed within the first 2 h after induction, accompanied by a maximum l‐valine excretion of 0.24 mmol_valine_ g_X_^–1^ h^–1^. The highest KIV consumption rate of 0.95 mmol_KIV_ g_X_^–1^ h^–1^ was measured in the micro‐aerobic *production phase II* in which isobutanol production rate (0.17 mmol_isobutanol_ g_X_^–1^ h^–1^) was still half of the maximum rate.

### 3.2 High growth rates lead to increased product and by‐product formation

With the aim to increase the biomass specific isobutanol production rate, fast cell growth of 0.22 h^–1^ that is close to μ_max_ was installed during the fed‐batch phase (from ‐10.9 to 0 h) in *Process F* by adjusting the glucose feed (see Figure 1). The plasmid encoded isobutanol production was started with 16.6 g_X_ L^–1^ biomass concentration (*t* = 0 h). Within 4 h of *production phase I*, 3.0 g_isobutanol_ L^–1^ accumulated. 2‐KG formation increased by 3.3‐fold, reaching a maximum medium concentration of 82 g_2‐KG_ L^–1^ that accounts for 0.58 g_2‐KG_ g_glucose_^–1^. Consequently, the total glucose consumption increased by 41% still revealing an almost equal isobutanol yield of 25 mg_isobutanol_ g_glucose_^–1^ compared to the reference *Process R* (Table 1). After reaching a peak at 18.3 g_X_ L^–1^ biomass concentrations declined by more than 20% (from 2 to 6 h). Switching to micro‐aerobic condition after 6 h only marginally affected isobutanol production. The maximum of 3.35 g_isobutanol_ L^–1^ in *production phase II* coincided with the depletion of the extracellular precursor KIV.

As illustrated in Figure 2, the high growth rate during fed‐batch in *Process F* resulted in a 1.7‐fold enhanced maximum isobutanol production rate of 0.65 mmol g_X_^–1^ h^–1^ in relation to the reference *Process R*. Noteworthy, within *production phase I* in *Process F*, glucose consumption rate almost tripled from 2.7 to 7.6 mmol_glucose_ g_X_^–1^ h^–1^ and 2‐KG formation rate increased by 24‐fold in contrast to the uninduced cells. Interestingly, despite an increased glucose supply and consumption in *Process F*, the combined carbon uptake of glucose, gluconate and 2‐ketogluconate did not exceed 2.4 mmol g_X_^–1^ h^–1^ although a maximum carbon uptake of approx. 8 mmol g_X_^–1^ h^–1^ was observed during the batch phase. Even more, the growth rate declined drastically by nearly 70% within the first 2 h of *production phase I* followed by a phase of reduced cell density. During production of isobutanol, up to 0.28 mmol_gluconate_ g_X_^–1^ h^–1^ was measured. Additionally, extracellular accumulated KIV was almost completely consumed during *production phase I*. To conclude, the set growth rate close to μ_max_ led to increased production of isobutanol but also to exorbitantly high oxidation of glucose to 2‐KG and a cessation of growth.

### 3.3 Metabolic constraints during aerobic overproduction of isobutanol

**FIGURE 3:**
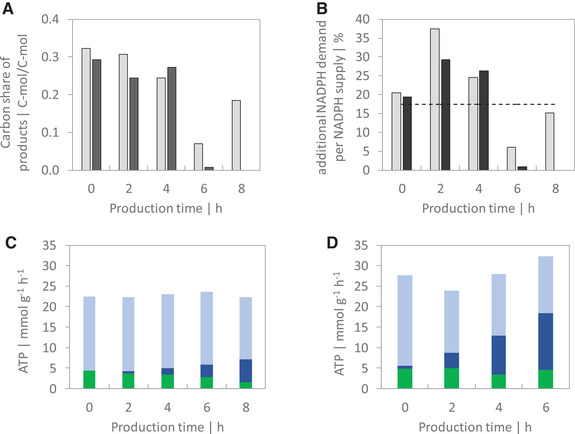
(A) Carbon share of the products KIV, l‐valine, isobutanol, and CO_2_ per uptake of substrates in *Process R* (grey bar) and *Process F* (dark grey bar). (B) NADPH demand for production of KIV, l‐valine and isobutanol as percentage of the total NADPH supply in *Process R* (grey bar) and *Process F* (dark grey bar). The dotted line represents the theoretical NADPH surplus in *P. putida* WT. (C‐D) Biomass specific ATP formation rates based on oxidative phosphorylation (q_ATP,O_, sum of dark blue and light blue bar) and substrate level phosphorylation (q_ATP,S_, green bar) in (C) the reference *Process R* and (D) the *Process F*. The dark blue bar represents the ATP generation based on the electron transport via oxidation of glucose to 2‐KG (q_ATP,alt_). The light blue bar represents the remaining part of oxidative phosphorylation via NADH and FADH_2_ (=q_ATP,O_ – q_ATP,alt_). The production time in (A‐D) is given with respect to induction of the plasmid whereas 0 h equals the reference condition before plasmid induction

As illustrated in Figure 3A, approximately 30% of the total carbon uptake during the fed‐batch phase in *Process R* and *Process F* accounts for the products KIV, isobutanol, l‐valine, and CO_2_ that are formed in the production pathway. Hereby, most of the carbon is excreted in form of KIV. During *production phase I*, the product per substrate carbon share in the isobutanol pathway is slightly decreased, but still above 20%. After 4 h of *production phase I*, a reduced isobutanol production rate resulted in less than 10% product formation per carbon uptake. Moreover, we calculated the additional NADPH that is consumed within the production pathway based on the product formation rates. The total NADPH supply was estimated from published NADPH production rates in *P. putida* KT2440 [21, 39]. The authors found that glucose‐grown *P. putida* consumes most of the created NADPH by anabolic reactions except of a surplus of 14 to 21%. As shown in Figure 3B, the NADPH surplus (17.4% as average of literature values) covers the additional NADPH demand from overexpressed enzymes during the fed‐batch phase. However, during the first 4 h of *production phase I* the additional NADPH demand exceeds the theoretical NADPH surplus which coincides with a decline of the growth rate in both processes.

Furthermore, we estimated the ATP formation of the cells based on their carbon and oxygen uptake. Additionally, the oxidation of glucose to gluconate and 2‐KG releases electrons that reduce the respiratory chain component PQQ [29, 41]. In return, the proton gradient created by the electron transport chain drives the ATP synthase. Thus, the periplasmatic oxidation of glucose to 2‐KG contributes to the overall ATP formation from oxidative phosphorylation. More precisely, Figures 3C,D illustrate the ATP formation from substrate level and oxidative phosphorylation before and after plasmid induction during aerobic production. The latter consists of electron transfers from NADH, FADH_2_, and periplasmatic glucose oxidation. The total ATP generation in *Process R* and *Process F* remains nearly constant during *production phase I*. Remarkably, the amount of ATP generated by electrons released from the 2‐KG pathway increased from 0.6 to 5.7 mmol g_X_^–1^ h^–1^ in *Process R* and from 3.8 to 13.9 mmol g_X_^–1^ h^–1^ in *Process F*, resulting in an ATP coverage of 50% at the end of *production phase I* in *Process F*.

**FIGURE 4:**
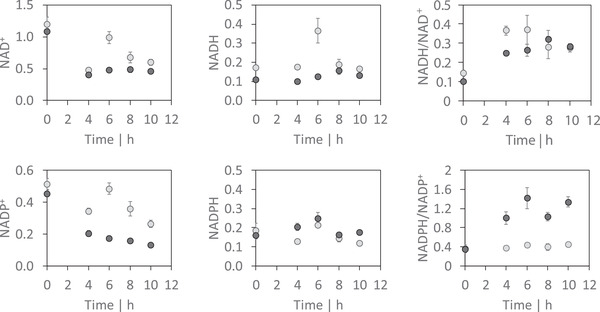
Intracellular concentrations (in μmol g_X _^–1^) of the redox cofactors NAD^+^, NADP^+^, NADH and NADPH and the respective redox ratios during the production phase in the reference *Process R* (grey circle) and in *Process F* (black circle). Time point 0 h equals the reference condition before induction of the plasmid

Figure 4 shows absolute concentrations [μmol g_X_^–1^] of intracellular redox cofactors NAD^+^, NADH, NADP^+^ and NADPH that were detected in the aerobic *Process R* and *Process F*. Reduced NAD(P)H analogues are often heavily degraded under standard cold‐based quenching/extraction conditions [43]. However, alkaline conditions (pH 9.2) with varying buffer concentrations and the addition of the reducing agent mercaptoethanol stabilized reduced pyridine nucleotides in solution [44]. Thus, by testing different buffer and reducing agents, we identified the supplementation of ammonium acetate to methanol as optimal quenching solution and supplementation of ammonium acetate plus 3‐mercaptopropioinic acid to methanol as ideal extraction solution in regard of reliable and stabilized NAD(P)H species. This method was suitable to detect oxidized as well as reduced nucleotides. Under aerobic isobutanol production in *Process R* and *Process F*, pools of intracellular oxidized cofactors NAD^+^ and NADP^+^ were found to be significantly decreased in *production phase I* and *phase II* (see Figure 4). Remarkably, the level of the reduced species NADH and NADPH remained nearly constant in both processes except for an NADH outlier after 6 h in *Process R*. Consequently, isobutanol producing *P. putida* show higher catabolic reduction charges (NADH/NAD^+^) in both processes and slightly enhanced anabolic reduction charge in *Process F*. Further intracellular central carbon metabolites are shown in Figure S2.

### 3.4 Micro‐aerobic conditions increased isobutanol yield and reduced by‐product formation

As shown in the reference *Process R*, switching from aerobic to micro‐aerobic condition significantly reduced the by‐product formation with almost unchanged product formation at the same time. Therefore, *Process MA* was performed similarly to *Process R*, but micro‐aerobic condition (DOT = 0%) was installed already 20 min after plasmid induction in *production phase I*. The results of this process were validated in a biological duplicate (refer to Table S1). The related process kinetics can be drawn from Figure 1. As expected, no extracellular accumulation of 2‐KG was observed in the *production phase I*. However, the biomass concentration declined considerably from 12.4 to 10.2 g_X_ L^–1^ during the micro‐aerobic phase. Moreover, extracellular accumulated KIV was completely converted to isobutanol within 6 h after induction, resulting in a maximum titer of 2.67 g_isobutanol_ L^–1^. This process strategy yielded 60 mg_isobutanol_/g_glucose_ within only 4 h, which is two times higher compared to *Process F* and *Process R*. Besides, total glucose consumed was 58% less than in the reference *Process R*. Furthermore, the extracellular by‐product l‐valine was approx. 50% lower than detected during both aerobic production strategies. As illustrated in Figure 2, only a minimal glucose consumption rate was observed during micro‐aerobic condition in *Process MA*. Therefore, carbon uptake into the cells was only approx. 10% of aerobic carbon uptake. Concomitantly, the growth rate turned negative resulting in reduction of cell concentration. However, nearly equimolar conversion of extracellular KIV into isobutanol was observed within 4 h of oxygen limited production in *Process MA*, but isobutanol production ceased after complete consumption of KIV. The Figure S3 illustrates the intracellular concentrations of carbon metabolites and redox cofactor during micro‐aerobic isobutanol production. Notably, succinic acid accumulated more than six‐fold during oxygen depletion compared to aerobic growth.

In addition, carbon balance revealed that 12.8% of glucose was fuelled into the l‐valine and the Ehrlich pathway, of which 9.2% accounts for isobutanol, in case of the optimized *Process MA* (Table 2). In contrast, only 4% of glucose was channelled into isobutanol during aerobic production in *Process R* and *Process F*. Even more, aerobic, fast‐growing *P. putida* cells (*Process F*) lost 58% carbon into 2‐KG which does only occur to a minimal extent (0.6%) under micro‐aerobic production.

**TABLE 2:** Carbon balance derived from the three different process strategies

Process strategy	Biomass	Isobutanol	2‐KG	CO_2_	Gluconate	2‐KIV	l‐valine	Sum
*Process R*	0.19	0.04	0.24	0.47	0.01	0.01	0.03	0.98
*Process MA*	0.28	0.09	0.01	0.57	0.01	0.01	0.04	1.00
*Process F*	0.12	0.04	0.57	0.24	0.01	0.00	0.02	1.01

Each value represents the carbon share (C‐mol/C‐mol) of the product based on the total glucose consumed at the end of the process.

## 4 DISCUSSION

### 4.1 NADPH limitation constrains the growth‐coupled isobutanol production in *P. putida*

**FIGURE 5:**
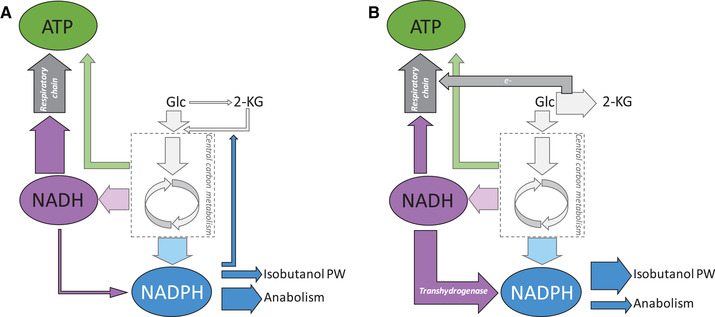
Schematic overview of assumed metabolic fluxes that are related to the intracellular pools of ATP, NADH, and NADPH (A) before induction and (B) during isobutanol formation in *P. putida* Iso2. The thickness of the arrows indicates the intensity of the related flux

The overexpression of the recombinant enzymes IlvD and YqhD to produce isobutanol obviously leads to an excessive demand of the cofactor NADPH in *P. putida* Iso2. This resulted in metabolic constraints affecting the cellular pools of NADPH, NADH and ATP which we depicted in Figure 5. As shown by Kohlstedt and Wittmann [39], the metabolism of *P. putid*a creates a surplus of NADPH in growing cells that is most likely converted into NADH by the transhydrogenase. Interestingly, isobutanol production in *P. putida* was only possible after knockout of the transhydrogenase *stha* [23]. This genetic manipulation enabled the ample supply of NADPH for the isobutanol production pathway. As shown in Figure 3, the cofactor surplus was sufficient to cover the overproduction of KIV and l‐valine during the non‐induced fed‐batch phase. The expression of the relative enzymes before induction most likely mirrors leaky plasmid expression. However, the overexpression of Yqhd after induction created a further NADPH sink that exceeded the NADPH surplus. As a result, NADPH demand in the production pathway competed with anabolic processes which resulted in reduced growth in *Process R*. In fact, a 66% higher production rate in *Process F* coincided with cessation of growth. Furthermore, glucose uptake is accompanied by partial periplasmatic oxidation to gluconate and to 2‐KG [21]. In contrast to recent approaches to prevent 2‐KG secretion by deletion of the glucose dehydrogenase *gcd* [23, 24, 25], the glucose limited feeding strategy inhibited extracellular secretion of 2‐KG before plasmid induction. However, after induction, the high NADPH demand in the isobutanol pathway competed with the NADPH dependent uptake of 2‐KG and may partially explain the extracellular accumulation of this organic acid. In addition, to satisfy the extensive NADPH demand, an increased activity of the membrane bound transhydrogenase PntAB is likely to occur. This enzyme's favoured reaction is the reduction of NADP^+^ at the expense of NADH via proton translocation [45, 46] which leads to a reduced availability of NADH for respiratory ATP generation. To satisfy the cellular ATP demand, electrons for the respiratory chain are released by periplasmatic oxidation of glucose to 2‐KG [29, 41] which decouples ATP generation from the availability of NADH or FADH_2_ as electron donor. This hypothesis is supported by the immense extracellular accumulation of 2‐KG during aerobic isobutanol production that resulted in a carbon loss of up to 58%. Concomitantly, the electron supply from periplasmatic glucose oxidation contributed to total ATP generation with rising significance (Figure 3). The estimated total ATP generation in *P. putida* Iso2 is close to the theoretical demand of 15 mmol_ATP_ g_X_^–1^ h^–1^ at *μ* = 0.13 h^–1^ and 24 mmol_ATP_ g_X_^–1^ h^–1^ at *μ* = 0.22 h^–1^ for wildtype *P. putida* [40]. The crucial role of periplasmatic glucose oxidation during NADPH intensive product formation in *P. putida* is underlined by the lacking isobutanol production in the *Δgcd* strain [23].

These results show that cells are endowed to quickly adapt NADPH supply and energy management to current needs. Figure 4 outlines that physiological NADH and NADPH levels could be maintained for the sake of lower NAD^+^ and NADP^+^ levels which finally increased catabolic and anabolic reduction states. Still, the resulting redox ratios are in good agreement with published findings measured in *P. putida* KT2440 [47, 48]. Since NADPH is also required to counteract oxidative [49] and chemical stress [50, 51], the cellular need to maintain physiological levels or even increase the cofactor supply through rearranging metabolic fluxes [42] is important. Accordingly, the NADPH requirements for isobutanol compete with the ‘life‐style’ of *P. putida* requiring for further systems metabolic engineering measures to succeed.

Even though the fed‐batch cultivation significantly increased the isobutanol titer compared to shake flask (0.12 g L^–1^) [23] and exceeds the product formation of engineered *Pseudomonas* sp. strain [52], the production efficiency of *P. putida* Iso2 is still not competitive with high yield aerobic or anaerobic approaches using, e.g. *E. coli* or *C. glutamicum* [3].

### 4.2 Oxygen limited stationary conditions benefit NADPH dependent isobutanol formation

*Processes R*, *F* and *MA* vary by the installation of different post‐induction aeration scenarios. In contrast to aerobic conditions that enabled growth‐coupled isobutanol formation, micro‐aerobic conditions limited cell growth leading to growth‐decoupled isobutanol production. The dual‐phase cultivation strategy, comprising aerobic biomass formation followed by micro‐aerobic isobutanol production in *Process MA* substantially improved the product yield and inhibited extracellular by‐product formation. Moreover, the micro‐aerobic process strategy optimized NADPH dependent product formation—within the cellular capacities as illustrated above—but hampered the growth of the strict aerobic microbe *P. putida*. The controlled limitation of oxygen supply forced the cells into a stationary state as their energy generation is mainly dependent on the electron transport chain with the final electron acceptor oxygen [39]. In this case, electrons cannot be transferred by the succinate dehydrogenase to reduce FAD^+^ which explains the observed intracellular accumulation of succinic acid that is seven‐fold higher in *production phase I* of *Process MA* compared to the pre‐induction level. In accordance, accumulation of succinic acid was also observed for anaerobic production of isobutanol in *C. glutamicum* [9]. Noteworthy, *P. putida* Iso2 showed a substantially higher volumetric isobutanol productivity of 9.5 mmol L^‐1^ h^–1^ during micro‐aerobic conditions compared to 4.4 mmol L^‐1^ h^–1^ observed for *C. glutamicum* [9]. Furthermore, the micro‐aerobic production condition in *Process MA* resulted in direct conversion of KIV into isobutanol which doubled the formation rate in contrast to *Process F* and reduced the product related NADPH costs. We estimated a lower additional NADPH demand of 1.1 mol_NADPH_ mol_isobutanol_^–1^ in *Process MA* compared to 1.5 mol_NADPH_ mol_isobutanol_^–1^ and 1.9 mol_NADPH_ mol_isobutanol_^–1^ in *Process R* and *Process F*, respectively. Nevertheless, metabolic activity of *P. putida* is a prerequisite for NADPH supply in a micro‐aerobic environment and could potentially be monitored using exhaust gas analysis [53]. In agreement with Lai et al. [29] and Nitschel et al. [23], minimal carbon uptake to eventually generate NADPH through the central enzymes Zwf, Icd and MaeB [21, 39] was detected during oxygen depletion. We estimated a total NADPH formation of 510 mmol_NADPH_ from carbon uptake versus a total demand of only 300 mmol_NADPH_ needed as cofactor for Yqhd to convert isobutyraldehyde into isobutanol during 4 h of micro‐aerobic production in *Process MA*. Thus, a sufficient amount of cofactors was supplied for the conversion of KIV into isobutanol in stationary *P. putida* cells.

To conclude, stationary cells and sufficient precursor supply qualifies micro‐aerobic isobutanol formation in *P. putida* Iso2 as more efficient in terms of energy and NADPH demand compared to aerobic production. Moreover, the formation of 2‐ketogluconic acid appears to be a unique trait in *P. putida* to balance its energy supply as a countermeasure to cofactor limitation. The novel findings of oxygen limited succinate accumulation could pave the way for innovative biotechnological applications with *P. putida*. Since micro‐aerobic isobutanol production only occurred until extracellular KIV was depleted, we assume a hampered carbon flux to the precursor KIV during oxygen depletion. This bottleneck and the role of the transhydrogenase PntAB should be addressed by subsequent metabolic engineering studies to facilitate NADPH demanding product formation with *P. putida* KT2440.

 

**Nomenclature**
*Y* [g g_glucose_^–1^]: Product yield per glucose consumed
*q*_i_ [mmol g_X_^–1^ h^–1^]: Biomass specific production rate of compound *i*

**Greek symbols**
*μ* [h^–1^]: Cell‐specific growth rate

**Indices**
X: Biomass
S: Substrate glucose
O: Oxygen
P: Product isobutanol

## CONFLICT OF INTEREST

The authors have declared no conflict of interest.

## Supporting information

Supplementary information

## Data Availability

The data that support the findings of this study are available from the corresponding author upon reasonable request.
