# Supplementary material for: Micro‐aerobic production of isobutanol with engineered Pseudomonas putida
Source: Eng Life Sci. 2021 Mar 13;21(7):475–88. doi: 10.1002/elsc.202000116 (PMC8258000; doi:10.1002/elsc.202000116)
Supplement: Supplementary file 1 — Supplementary information [file ELSC-21-475-s001.pdf]

Supplementary file

Micro-aerobic production of isobutanol with engineered *Pseudomonas putida*

Andreas Ankenbauer[1]

Robert Nitschel[1]

Attila Teleki[1]

Tobias Müller[1]

Lorenzo Favilli[1]

Bastian Blombach[2]

Ralf Takors[1]

[1]Institute of Biochemical Engineering, University of Stuttgart, Allmandring 31, 70569 Stuttgart, Germany

[2]Microbial Biotechnology, Campus Straubing for Biotechnology and Sustainability, Technical University of Munich, Straubing, Germany

Correspondence: Prof. Ralf Takors (takors@ibvt.uni-stuttgart.de). Institute of Biochemical Engineering, University of Stuttgart, Allmandring 31, 70569 Stuttgart, Germany.

Supplementary Process Characteristics

Fehler! Verweisquelle konnte nicht gefunden werden. shows the extracellular glucose concentrations, the dissolved oxygen tensions, and the Feed rates in the *Process R*, *Process F*, and *Process MA*. Table S 1 lists the process related results from the biological duplicate of *Process MA*.

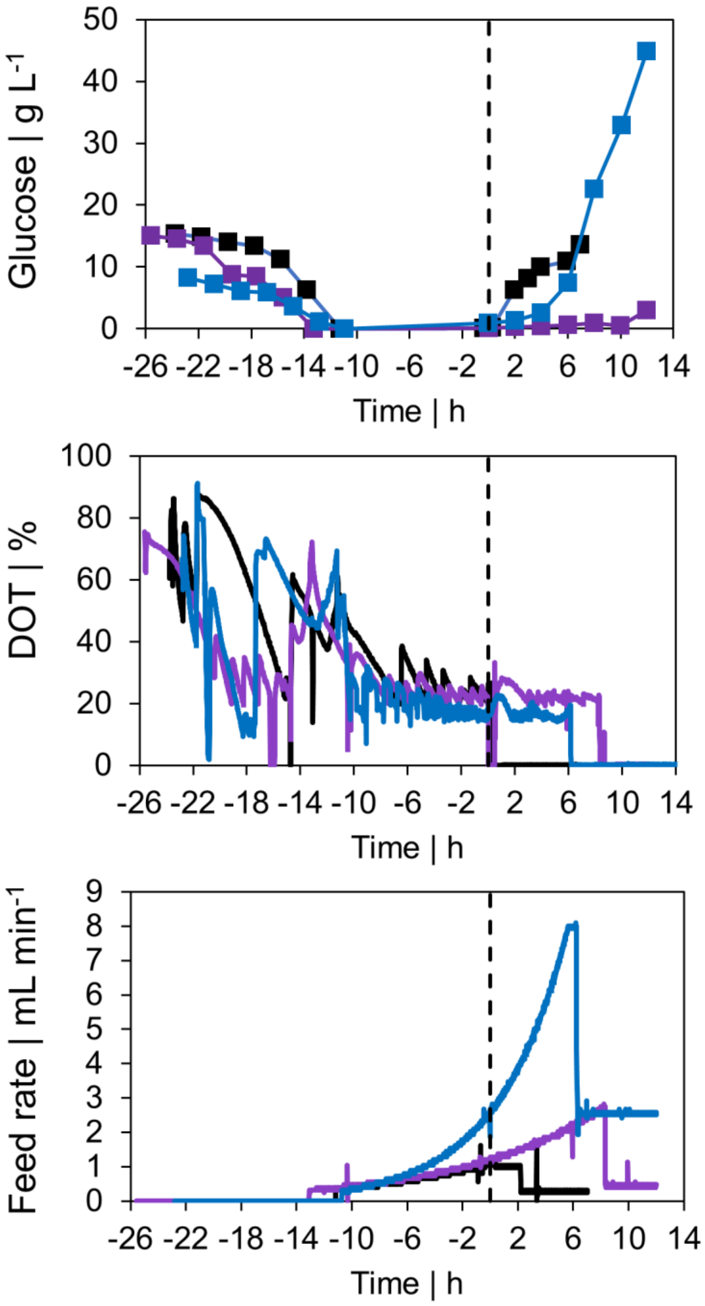

Figure S 1 Extracellular glucose concentrations, dissolved oxygen tensions (DOT), and the feed rates in Process R (purple), Process F (blue), and Process MA (black)

**Time**		**Biomass**		**Isobutanol**		**2-KG**		**KIV**		**L-valine**		**Y_{PS}**		**DOT**	
[h]		[g L^{-1}]		[g L^{-1}]		[g L^{-1}]		[g L^{-1}]		[g L^{-1}]		[mg g^{-1}]		[%]	
-24.1	-23.8	0.1	0.1	0.0	0.0	0.2	0.1	0.1	0.1	0.0	0.0	0.0	0.0	41.7	66.8
-22.1	-21.8	0.1	0.1	0.0	0.0	0.4	0.2	0.1	0.1	0.0	0.0	0.0	0.0	60.2	52.8
-20.1	-19.8	0.2	0.2	0.0	0.0	0.9	0.7	0.2	0.1	0.0	0.0	0.0	0.0	48.1	79.0
-18.1	-17.8	0.5	0.3	0.0	0.0	1.7	1.5	0.3	0.2	0.0	0.0	0.0	0.0	49.6	58.3
-16.1	-15.8	1.0	0.8	0.0	0.0	3.3	2.8	0.4	0.4	0.0	0.0	0.0	0.0	58.0	31.8
-14.1	-13.8	1.9	1.3	0.0	0.0	5.3	4.9	0.9	0.6	0.0	0.0	0.0	0.0	33.0	54.2
-13.3	-11.3	2.4	2.7	0.0	0.0	5.9	9.8	0.9	1.1	0.1	0.0	0.0	0.0	60.3	56.0
0.0	-0.4	12.5	12.4	0.1	0.1	0.5	0.0	6.4	7.3	1.0	0.8	2.1	2.2	26.3	22.7
0.3	0.3	12.5	12.1	0.1	0.1	0.5	0.0	6.3	7.4	1.1	0.9	2.5	2.9	24.1	26.6
2.0	1.9	11.2	11.6	1.5	1.5	0.5	0.0	4.2	1.9	1.2	1.1	20.5	32.9	0.1	0.0
3.0	3.0	11.1	11.1	1.9	2.3	0.3	0.2	1.0	0.7	1.2	1.1	42.7	52.0	0.0	0.3
4.0	3.9	10.6	11.0	1.9	2.7	0.3	0.2	0.4	0.3	1.3	1.1	55.6	59.7	0.0	0.2
6.0	5.9	10.2	10.5	2.6	2.6	0.4	0.2	0.2	0.0	1.1	1.0	49.4	59.2	0.5	0.1
8.0	6.9	10.2	10.2	2.5	2.6	0.4	0.3	0.0	0.0	1.1	1.0	49.0	58.8	0.3	0.1

Table S 1 Comparison of relevant product concentrations and yields that were determined in the biological duplicate experiment of Process MA. The process time is given in relation to the point of plasmid induction. The grey columns represent the results shown in the manuscript.

Intracellular metabolite quantification

Figure S 2 illustrates intracellular metabolite levels that were determined before and during aerobic isobutanol production in *P. putida* Iso2.

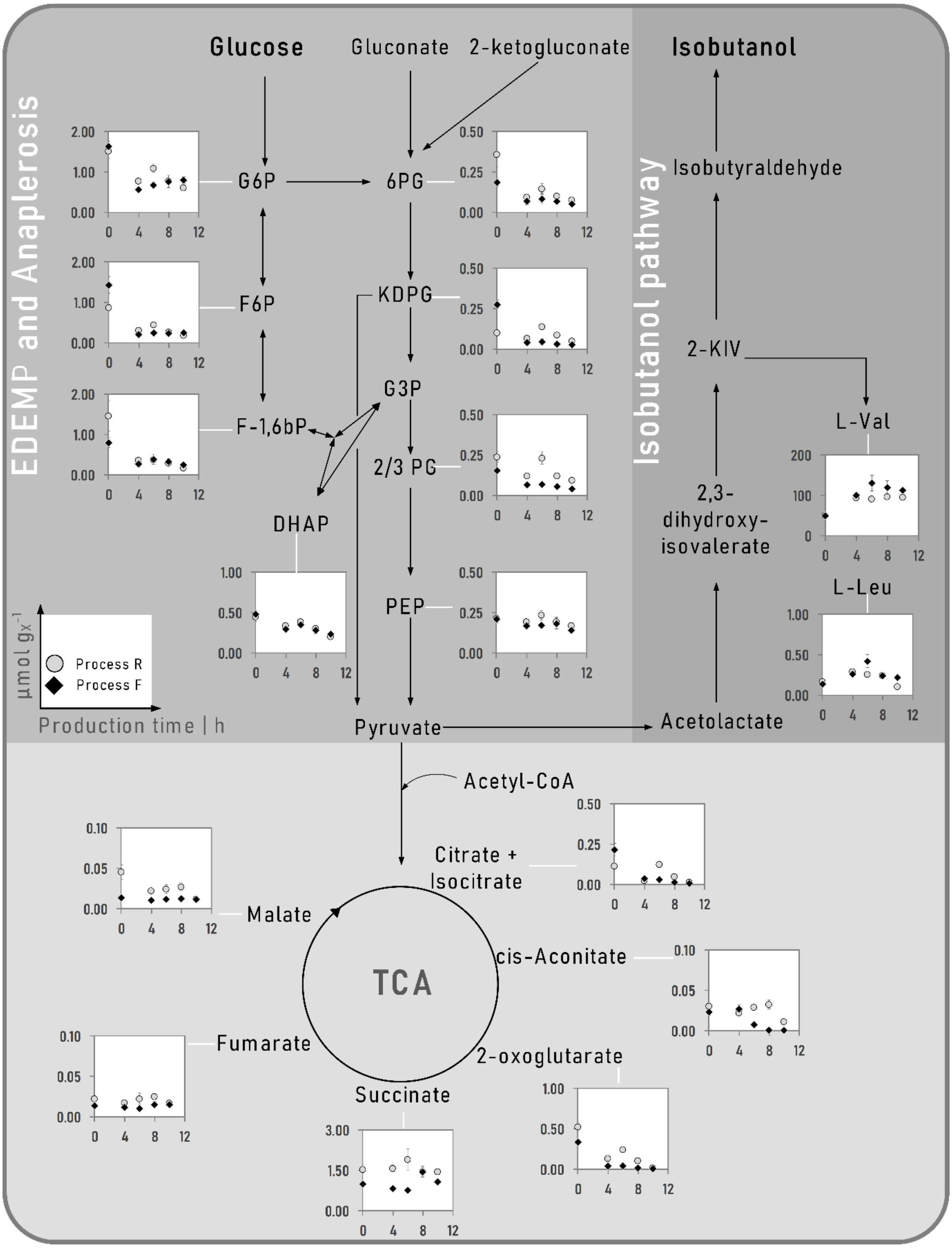

Figure S 2 Intracellular concentrations (in µmol g_X^{-1}) of different metabolites in isobutanol producing P. putida Iso2 cells under aerobic fed-batch conditions in the reference Process R (⭘) and Process F (◆). Time point 0 h equals the reference

condition before induction of the plasmid. Micro-aerobic condition was installed after 8 h in Process R and after 6 h in Process F, respectively. Abbreviations: G6P: glucose-6-phosphate, 6PG: 6-phosphogluconate, KDPG: 2-keto-3-deoxy-6-phosphogluconate, G3P: glyceraldehyde-3-phosphate, 2/3 PG: 3-phosphoglycerate and 2-phosphoglycerate, PEP: phosphoenolpyruvate, DHAP: dihydroxyacetone-phosphate, F-1,6-bP: fructose-1,6-bisphosphate, F6P: fructose-6-phosphate, CoA: co-enzyme A, TCA: tricarboxylic acid cycle, L-Leu: L-leucine, L-Val: L-valine, 2-KIV: 2-ketoisovalerate

Figure S 3 shows intracellular metabolite concentrations after 4 hours of micro-aerobic condition in contrast to the aerobic level before plasmid induction in *Process MA.*

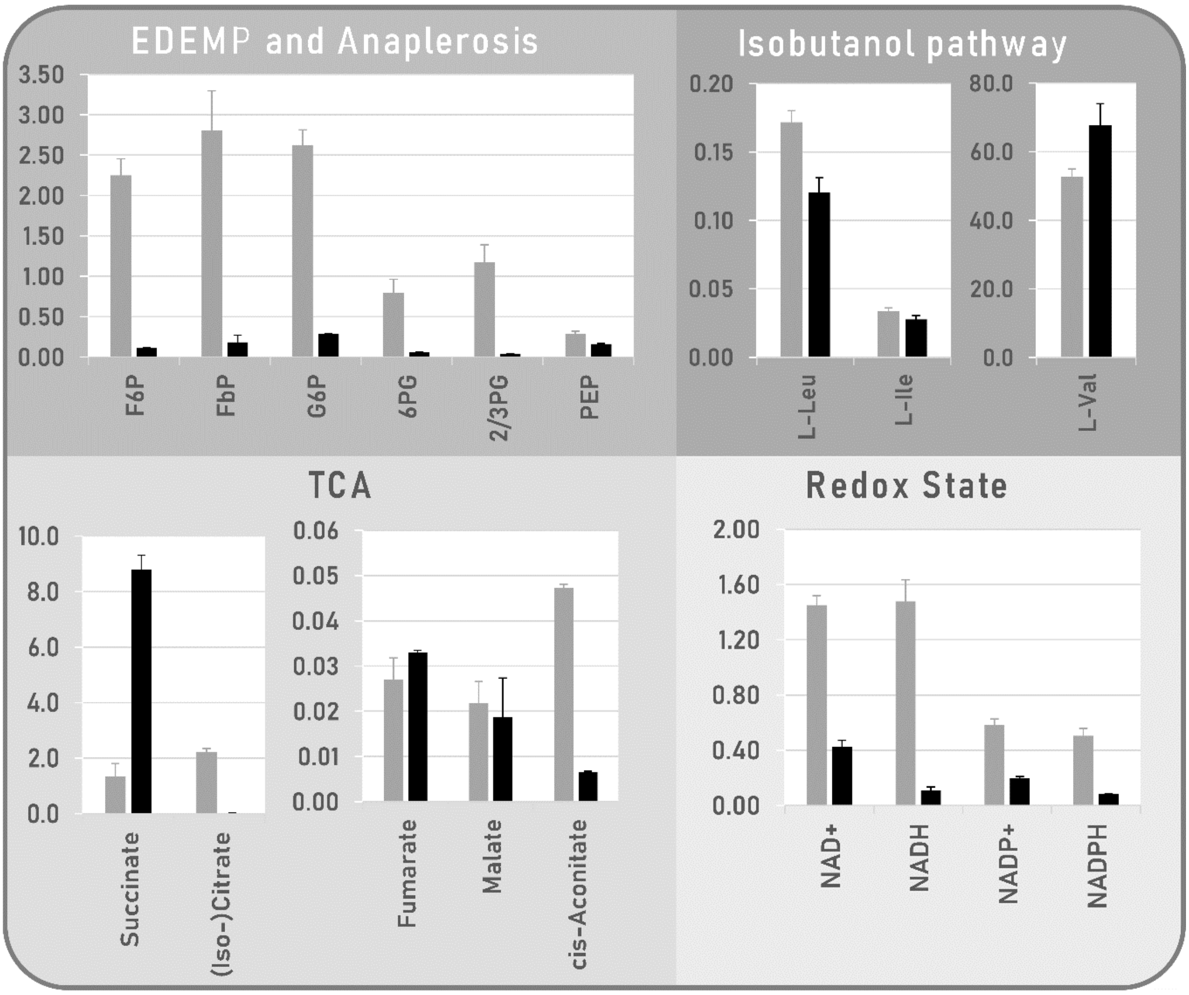

Figure S 3 Comparison of intracellular concentrations (in µmol g_X^{-1}) of different metabolites in isobutanol producing P. putida Iso2 cells under micro-aerobic condition (black bars) in contrast to aerobic growing cells (grey bars) in Process MA. Abbreviations as addition to Figure S1: L-Ile: L-isoleucine
